# High accuracy thermal conductivity measurement of aqueous cryoprotective agents and semi-rigid biological tissues using a microfabricated thermal sensor

**DOI:** 10.1038/srep10377

**Published:** 2015-05-20

**Authors:** Xin M. Liang, Praveen K. Sekar, Gang Zhao, Xiaoming Zhou, Zhiquan Shu, Zhongping Huang, Weiping Ding, Qingchuan Zhang, Dayong Gao

**Affiliations:** 1 Centre for Biomedical Engineering, Department of Electronic Science and Technology, University of Science and Technology of China, Hefei, Anhui 230027, China; 2 USTC Center for Micro- and Nanoscale Research and Fabrication, University of Science and Technology of China, Hefei, Anhui 230027, China; 3 Department of Mechanical Engineering, University of Washington, Seattle, WA 98195, USA; 4 School of Mechanical, Electronic, and Industrial Engineering, University of Electronic Science and Technology of China, Chengdu, Sichuan 611731, China; 5 Department of Biomedical Engineering, Widener University, Chester, PA 19013, USA; 6 CAS Key Laboratory of Mechanical Behavior and Design of Material, Department of Modern Mechanics, University of Science and Technology of China, Hefei, Anhui 230027, China

## Abstract

An improved thermal-needle approach for accurate and fast measurement of thermal conductivity of aqueous and soft biomaterials was developed using microfabricated thermal conductivity sensors. This microscopic measuring device was comprehensively characterized at temperatures from 0 °C to 40 °C. Despite the previous belief, system calibration constant was observed to be highly temperature-dependent. Dynamic thermal conductivity response during cooling (40 °C to –40 °C) was observed using the miniaturized single tip sensor for various concentrations of CPAs, i.e., glycerol, ethylene glycol and dimethyl sulfoxide. Chicken breast, chicken skin, porcine limb, and bovine liver were assayed to investigate the effect of anatomical heterogeneity on thermal conductivity using the arrayed multi-tip sensor at 20 °C. Experimental results revealed distinctive differences in localized thermal conductivity, which suggests the use of approximated or constant property values is expected to bring about results with largely inflated uncertainties when investigating bio-heat transfer mechanisms and/or performing sophisticated thermal modeling with complex biological tissues. Overall, the presented micro thermal sensor with automated data analysis algorithm is a promising approach for direct thermal conductivity measurement of aqueous solutions and soft biomaterials and is of great value to cryopreservation of tissues, hyperthermia or cryogenic, and other thermal-based clinical diagnostics and treatments.

With rapid advancement in modern bioscience and medicine, research on biological heat transport has received major attention over the years^1^,^2^,^3^,^4^,^5^,^6^,^7^,^8^,^9^. It is well accepted in modern medicine that temperature history and cooling/warming rates experienced by biomaterials during exothermic/endothermic procedures greatly affect the biological outcome. Accurate knowledge of thermal conductivity of various sized bio-specimens in a timely manner is not only essential to study thermal transport characteristics and mechanisms in biological tissues, but also pivotal in numerous clinical therapeutic applications, such as improving the detection accuracy in disease diagnosis, lowering the risk of killing nearby healthy tissue in hyper-/hypothermia for cancer treatment, and optimizing the survival rate in cryopreservation for organ transplantation^1^,^5^,^6^,^10^,^11^,^12^. Therefore, the central engineering task associated is to readily and accurately measure thermal conductivities of biologically relevant materials, such as CPAs and soft tissues, in a wide range of temperature domains.

Although there have been a number of approaches to characterize thermal conductivity, the majority of currently available direct measuring approaches are often challenging in many situations due to practical measurement constrains, such as differences in scale (sensor and sample are incompatible in size), material (sensors cannot accommodate corrosive or conductive liquids), state (sensors do not have adequate response time for non-equilibrium transient thermal events) and instrumentation (sensors are often home-made with poor device reliability and low production rate). For example, the steady state longitudinal heat flow (guarded hot plate) method^13^,^14^,^15^ obtains thermal conductivity data at steady state using a 1-D heat flow model along with the knowledge of the heat flow and temperature gradient^3^, which requires cumbersome experimental apparatus, extensive specimen preparation, and long hours of measurement duration necessary for analysis^1^,^3^,^6^. A number of transient techniques, such as the thermal comparator method^16^,^17^, the self-heated thermistor method^18^,^19^,^20^,^21^,^22^,^23^, and the heated thermocouple method (similar variations including hot wires and thermal needles)^1^,^4^,^7^,^8^,^16^,^24^,^25^,^26^,^27^,^28^,^29^,^30^, have also been developed and widely used for measuring thermal conductivities and thermal diffusivities of various materials. Although these transient technique based traditional thermal conductivity sensors offer numerous advantages such as elimination of convective error, high accuracy and faster experimental response, drawbacks such as lack of configurable sensor geometries, necessity of labor-intense individual sensor calibration due to significant sensor-to-sensor variation in performance, difficulty in sensor fabrication, and severe localized material property alteration due to the insertion of a macroscopic sized foreign object with a large amount of heat generation^1^,^3^,^6^, have greatly reduced the usefulness of the developed sensors. Therefore, ongoing efforts have been focused on the development of miniaturized devices for biologically relevant materials. So far, among the few reported attempts in reducing the size of thermal conductivity sensors^6^,^9^,^31^,^32^,^33^,^34^,^35^, only two^6^,^9^ were capable of obtaining thermal conductivity information for biological samples while the others can only be used for aqueous materials. Despite the millimeter-ranged sensor size and intense manual post-experiment data processing, Liang and Yi’s microfabricated thermal conductivity sensors have demonstrated excellent sensor capabilities, such as easy to reconfigure and mass-produce, as well as high data accuracy and reliability for aqueous and biological materials.

In this study, with an aim to establish a standardized testing protocol for directly measuring the thermal conductivity of various solutions and soft biomaterials, we describe the development of an improved transient hot-wire (THW) based micro thermal conductivity sensor with fully automated post-experiment data analysis capability. This miniaturized device utilizes a SiO_2_/Au/SiO_2_ sandwiched structure to protect the microfabricated serpentine gold coil, which functions as both a heater and a passive thermometer for measuring the temperature response of the sample. Distilled water was used as the thermal standard material to obtain the temperature-specific system calibration constants at temperatures from 0 °C to 40 °C and to compare the performance characteristics across different sensors. To demonstrate the potential of the presented microdevice, series of experiments were conducted using first, the single-tip sensors to investigate the temperature effect on thermal conductivity of common biologically relevant CPAs, such as 1.5 M and 40% (W/V) G, 1.5 M EG and 10% (V/V) DMSO; and second, the multi-tip arrayed sensors (four identical probes) to study localized thermal conductivity variation of biological tissues with unknown degrees of anatomical heterogeneity, namely chicken breast, chicken skin, lean porcine limb and bovine liver.

## Results and discussion

### Micro thermal conductivity sensor system calibration using thermal standard materials

The presented thermal conductivity sensor is a miniaturized self-heated thermistor, which utilizes the standard THW measurement approach to determine the sample’s thermal conductivity based on the transient temperature response of the heating element^6^,^25^,^29^,^30^. In order to achieve the claimed high data accuracy, system calibration is still required for all THW based thermal conductivity sensors. However, as for the macroscopic counterparts, the necessity of labor-intense individual sensor calibration has greatly limited the practicality of these devices. Furthermore, up until now all measured thermal conductivities were obtained under the assumption that ambient testing temperature has insignificant influence on system calibration constant. The validity of such an assumption remains to be assessed. Therefore, to obtain the system calibration constant and evaluate its stability across different microfabricated sensors and under various ambient temperature conditions for the microfabricated thermal conductivity sensors, distilled water was used as the thermal standard fluid to calibrate three randomly selected single tip sensors at 0 °C, 5 °C, 10 °C, 20 °C and 40 °C.

**Figure 1:**
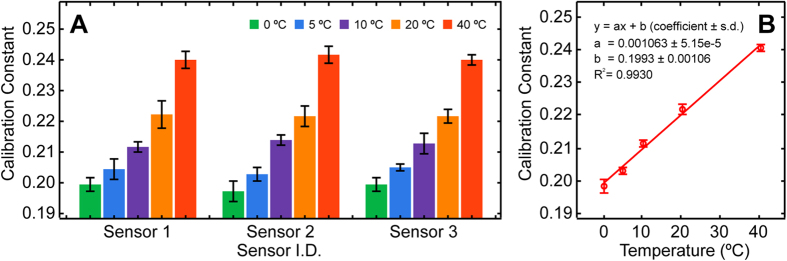
System calibration and performance evaluation. **(A)** System calibration constant determination using distilled water as the thermal standard liquid at 0, 5, 10, 20 and 40 ºC. Data represent mean ± standard error of the mean (S.E.M.) of five repeated measurements. **(B)** Linear response curve of system calibration constant with regard to ambient testing temperature variation. Data represent mean ± S.E.M. of all fifteen repeated experiments per temperature condition.

As illustrated in Fig. 1A, all sensors have exhibited excellent performance similarity in measuring thermal conductivity with a maximum percentage difference of 1.01% between different sensors. It was also evident that all sensors responded comparably when subjected to various ambient temperature conditions. Two-way ANOVA tests have revealed that the system calibration constant exhibited no statistical difference between sensors (*p* = 0.9901) and strong significant dependence to testing temperature (*p* < 0.0001). Our statistical analysis results have indicated that the microfabricated thermal conductivity sensors share high resemblance in terms of structural and performance characteristics, hence calibration constant obtained by one sensor can be applied to any sensor fabricated from the same batch. However, despite the generally accepted assumption that the system calibration constant depends solely on internal factors such as sensor geometry^6^,^9^,^24^,^25^,^27^,^29^,^30^, our results have suggested that such assumption is invalid and that external factor, like ambient testing temperature, also plays an important role affecting the system calibration constant. This finding led to the postulation that the use of a temperature-independent constant system calibration constant might substantially impact the subsequent thermal conductivity calculations. Therefore, by averaging the obtained thermal conductivity data according to the ambient testing temperatures, a linear trend between calibration constant and ambient temperature was observed (Fig. 1B). Temperature-specific calibration constants were then calculated based on the fitted linear function and used in all following measurements to ensure the highest possible data accuracy.

### Influence of temperature on thermal conductivity of CPA mixtures

Given that CPAs are widely used in cryopreservation and usually subjected to a wide range of temperatures, understanding how temperature affects their thermal properties can provide additional critical information in developing optimized cooling procedures to improve cell survival rate. The presented single tip sensors were randomly selected and employed to assess the temperature effect on thermal conductivities of four frequently used CPA mixtures viz. 1.5 M and 40% (W/V) glycerol (G), 1.5 M ethylene glycol (EG) and 10% (V/V) dimethyl sulfoxide (DMSO) at temperatures between −40 °C to 40 °C.

A representative electrical resistance over logarithmic time graph can be obtained in Fig. S-2. It was evident that the typical increase in the sensor’s electrical resistance throughout the experiment was approximately 0.02 Ω. Given the sensor is fundamentally a heated thermocouple, it can be concluded that the sensor induced local temperature elevation within the measured sample in a complete measurement was less than 0.01 °C. Therefore, it is reasonable to believe that the presented sensor does not significantly alter the thermal property of the measured sample and is able to deliver true and accurate thermal conductivity readings for aqueous CPAs and biological materials at a specific temperature.

**Figure 2:**
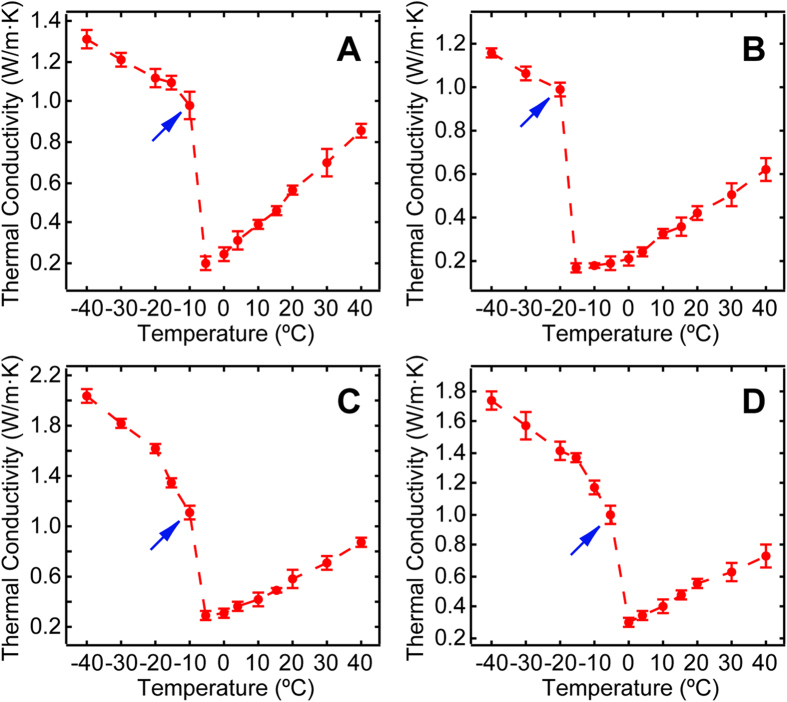
Dynamic thermal conductivity response to temperature variation for common biologically relevant CPA mixtures: **(A)** 1.5 M G. **(B)** 40% (W/V) G. **(C)** 1.5 M EG. **(D)** 10% (V/V) DMSO. Data represent mean ± 10 × S.E.M. of five independent measurements. Blue arrows denote the freezing points of each solution.

As shown in Fig. 2, the overall thermal conductivity response to temperature for these CPA mixtures can be divided into two sections: an initial quadratic decrement as the temperature drops, followed by a secondary rapid non-linear increment when the temperature continues to decrease and the solution completes its phase change (*p* < 0.0001, ANOVA). For −30 °C and −40 °C, thermal conductivity increased by an increment that was still statistically significant (*p* < 0.0001, Tukey’s post-hoc test), suggesting saturation in thermal conductivity was not reached. A maximum variation of 0.00795 W m^−1^ K^−1^ (S.E.M.) between trials was recorded indicating excellent data reliability. The observed substantial change in thermal conductivity agrees well with previous theoretical prediction and experimental results^3^.

**Figure 3:**
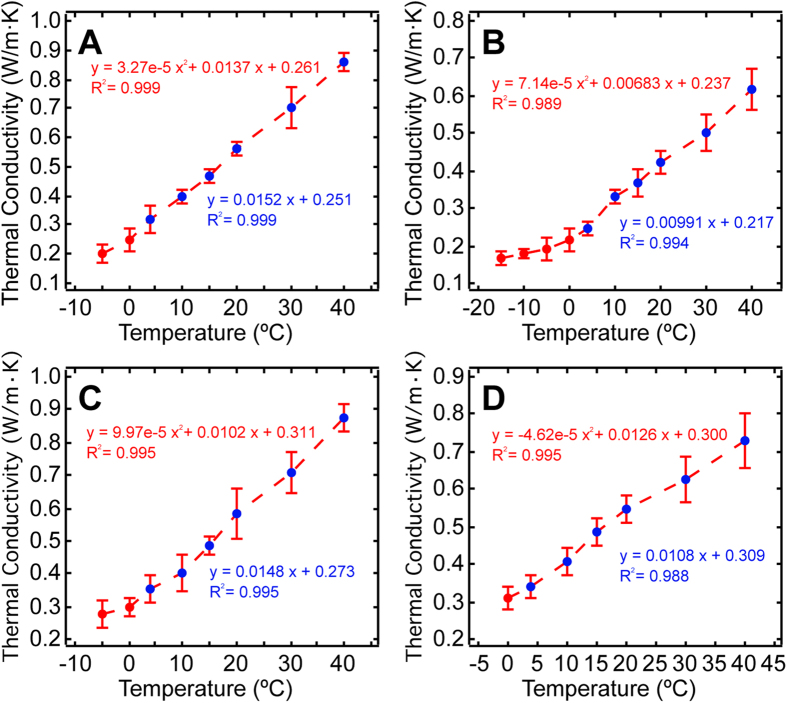
Magnified view of the unfrozen region of the dynamic thermal conductivity response to temperature variation graph (Fig. 2): **(A)** 1.5 M G. **(B)** 40% (W/V) G. **(C)** 1.5 M EG. **(D)** 10% (V/V) DMSO. Data represent mean ± 10 × S.E.M. of five independent measurements. Regions that exhibit strong linearity are labeled with blue dots.

It was also evident that despite the unique dynamic behavioral responses to temperature variation in the unfrozen region (before the completion of phase change), 10% V/V DMSO exhibited a convex shaped curve, while the rest of the solutions showed similar concaved response pattern (Fig. 3). Given that the measurement system is highly sensitive, it is our working hypothesis that such distinctive phenomenon is due to a higher degree of dissimilarity between DMSO with the other three CPAs at the molecular level within this temperature regime. However, a more comprehensive study from the materials science direction is needed before any definitive conclusions can be made. Furthermore, as illustrated in Fig. 3, there appeared to be a common linear region, from 4 °C to 40 °C, for all CPA mixtures, in which thermal conductivity increased monotonically with elevated temperatures. Measurement at 4 °C was specifically included, as it marks the anomalous expansion of water in cryobiology^36^. It seems that the change of water density due to water’s irregular expansion behavior also plays a significant role affecting the mixtures’ thermal property.

### Thermal conductivity distribution of soft biological materials

To better understand the thermal conductivity distribution for biological materials with unknown degree of anatomical homogeneity, a multi-tip arrayed micro thermal sensor (four identical probes) with a 150 μm probe-to-probe spacing was developed. Since each tip shared the same structural and electrical characteristics as the above described single tip sensor, the same system calibration constant relation with temperature was used in all multi-tip engaged measurements.

**Figure 4:**
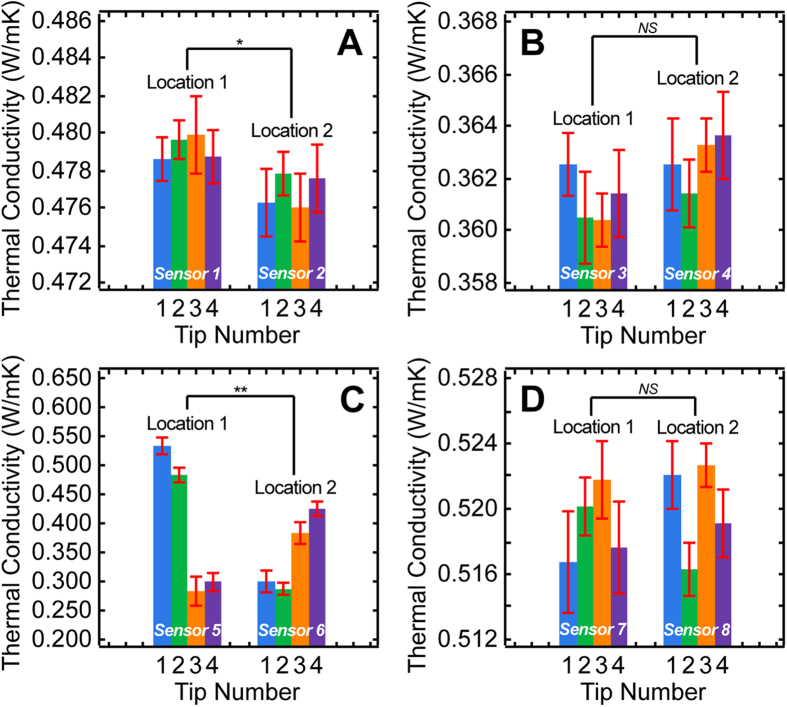
Thermal conductivity measurements for soft biological materials at 20 ºC using multi-tip arrayed micro thermal sensor. Localized thermal conductivity distribution of **(A)** Chicken breast. **(B)** Chicken skin. **(C)** Lean porcine limb. **(D)** Bovine liver. Data represent mean ± S.E.M. for (A), (B), (D), and mean ± 10 × S.E.M. for (C) of five repeated experiments. *NS:* *P* > 0.05, *: *P* < 0.05 and **: *P* < 0.01.

Two multi-tip arrayed sensors were randomly selected to perform thermal conductivity measurements at two arbitrary locations (at least 10 mm apart) per the following fresh biomaterials, chicken breast, chicken skin, lean porcine limb and bovine liver, at 20 °C. As illustrated in Fig. 4A, it was evident that there was a significant difference in thermal conductivity measurements between the two measurement locations (p = 0.0461, two-way ANOVA) and no statistical significant variation between individual tips of the multi-tip arrayed micro sensor (*p* = 0.872, two-way ANOVA). The results suggest that although chicken breast demonstrated high degree of localized homogeneity, thermal conductivity of such biomaterial did vary significantly at the macroscopic level. For chicken skin, our experimental results (Fig. 4B) showed statistically insignificant variation to both measurement location and individual sensing tips (*p* > 0.151, two-way ANOVA), which indicates chicken skin has a higher level of homogeneity at both local and tissue-wide scale. For lean porcine limb, given that the elevated anatomical complexity and heterogeneity, where swine muscle fibers interlace with adipose tissues, strong significant statistical dependence to both measurement location and individual sensing tips (*p* < 0.0001, two-way ANOVA) were observed (Fig. 4C). As shown in Fig. 4D, no statistically significant variation to both measurement location and individual sensing tips (*p* > 0.235, two-way ANOVA) was observed, which implies liver tissue is extremely homogeneous at both micro- and macroscopic level. A maximum run-to-run variation of 0.00493 W m^−1^ K^−1^ (S.E.M.) corroborates that the entire measurement system was highly stable in measuring thermal conductivity of biological samples. The observed strong correlation between thermal conductivity and physiological heterogeneity is likely due to substantial localized cellular composition difference.

Moreover, by averaging the measurements obtained from all tips and locations, the overall thermal conductivities for bulk chicken breast, chicken skin, lean porcine limb and bovine liver were estimated to be 0.478 ± 0.000548 W m^−1^ K^−1^, 0.362 ± 0.000498 W m^−1^ K^−1^, 0.375 ± 0.0147 W m^−1^ K^−1^, and 0.520 ± 0.000778 W m^−1^ K^−1^, respectively. In comparison with literature suggested values, the percentage differences for these biomaterials were found to be −8.03% (0.518 W m^−1^ K^−1^ at 25 °C^37^), 1.39% (0.357 W m^−1^ K^−1^ at 24 °C ~ 38 °C 14), −30.5% (0.510 W m^−1^ K^−1^ at 27 °C 6) and 3.92% (0.50 W m^−1^ K^−1^ at 25 °C 4). Despite the fact the newly determined average values are congruent with previous findings in general, there appear to be significant discrepancies between our results and the literature suggested values for chicken breast and porcine limb. Such disagreement is very likely due to inequitable biological samples and differences in measuring conditions. It is also evident that by averaging the thermal conductivities for a bulk measurement, the opportunities in obtaining more representative thermal conductivity distribution with respect to localized physiological composition variation for complex bio-tissues are lost. The developed multi-tip arrayed micro thermal conductivity sensor was proven to be capable of recognizing localized minor variations in thermal property, allowing a more realistic thermal conductivity distribution to be mapped. Taken together, these may provide deeper insight in improving our limited comprehension of the relationship between biophysics of biomaterials and their thermal properties.

## Conclusions

Motivated by the importance of having an accurate, reliable and convenient thermal conductivity measurement device for various soft biological materials and aqueous solutions, we have developed a new sensor system to readily measure their thermal properties. Due to the utilization of modern microfabrication, temperature-specific calibration constants, standardized testing protocol and automated MATLAB-based data analysis routine, the developed micro thermal conductivity sensor system significantly outperform its conventional counterparts in terms of data accuracy and reliability, device stability and applicability for a wider range of measuring conditions. The ease in microfabricating sensors with various sizes and shapes at both micro- and macro-scale on the same silicon wafer also makes our approach capable of mass-producing the presented thermal conductivity sensor to accommodate a variety of aqueous and biological samples. Moreover, because the MEMS based micro sensors share remarkable resemblance in terms of structural and electrical characteristics, system calibration process is greatly simplified, allowing the realization of calibration constants obtained by one sensor to be applied for an entire batch of thermal conductivity sensors.

Distinctive dynamic thermal conductivity responses during cooling (40 °C to –40 °C) were observed using the miniaturized single tip sensor for common CPA mixtures, i.e., 1.5 M and 40% (W/V) glycerol, 1.5 M ethylene glycol and 10% (V/V) dimethyl sulfoxide. Furthermore, since the microfabricated thermal conductivity sensor was powered by a constant current of 1 mA and the typical measurement run-time was 40 sec, the device induced specimen temperature elevation was less than 0.01 °C, which permits unaltered thermal property to be obtained without any artificial effect interference. It was possible for the first time to quantitatively realize the localized subtle thermal conductivity variation to tissue’s anatomical heterogeneity with a 150-μm resolution.

In summary, the presented minimally invasive micro thermal conductivity sensor along with the standardized experimental and data analysis protocol has enabled convenient and precise thermal conductivity measurements of various penetrable materials. Sensors with distinctive structural characteristic, like the arrayed multi-tip, can be used to accurately obtain the thermal conductivity of amorphous soft materials and study the role of intrinsic anatomical heterogeneity in biotissues as it is related to local thermal conductivity variation at the microscale. The developed micro thermal conductivity sensor approach has great potential in extracting thermal properties of non-rigid biomaterials and other important systems with or without cryoprotective agents in a wide range of temperature domains, especially in the cryogenic regime, insights gained with further investigations using the presented microscale thermal conductivity sensor may lead to the realization of better designs and more effective treatment strategies for destroying biological materials including tumors in cryosurgery.

## Methods

### Reagents

Photoresist AZ 1512 and AZ 1529 were obtained from AZ electronic materials, U.S.A. Glycerol, ethylene glycol, dimethyl sulfoxide and sodium chloride (NaCl) were obtained from Sigma Aldrich, U.S.A. Isotonic saline contained 0.9% w/v of sodium chloride. Unless otherwise stated, all CPA mixtures were prepared by dissolving pure CPAs in isotonic saline. Fresh biological tissues, namely chicken breast, chicken skin and lean porcine limb, were acquired from a local grocery store in Seattle, U.S.A.

### Measurement system

The schematic layout of the measurement system is shown in Fig. S-1A. The experimental apparatus consists of a presented micro thermal conductivity sensor, a Keithley Integra Series 2700 digital multimeter (DMM), a computer with data acquisition software, and a temperature regulated thermostatic bath. The electrical resistance response of the microfabricated sensing element over time was the primary measurement for the method. When measuring its electrical resistance changes, a small constant and continuous testing current of 1 mA ± 5% is applied to the circuit and the DMM records the voltage response cross the micro sensor, and then converts voltage into resistance using Ohm’s Law. For measurements using the four-tip probe, a manual switch and a simple breadboard circuit were added to the apparatus for switching the testing current sequentially to different tips. As demonstrated in previous work^6^, using a high precision DMM as both the constant current source and the digital ohmmeter, not only keeps the testing circuitry in its simplest form, but also minimizes system instability by requiring the least number of junctions to connect all components. The thermostatic bath (Jia Chuang, model HH-1) is used to provide an isothermal testing microenvironment for the micro thermal conductivity sensor of the measurement apparatus. The temperature regulatory function is automatically performed with a temperature perturbation that is less than 0.5 °C.

### Sensor fabrication

**Figure 5:**
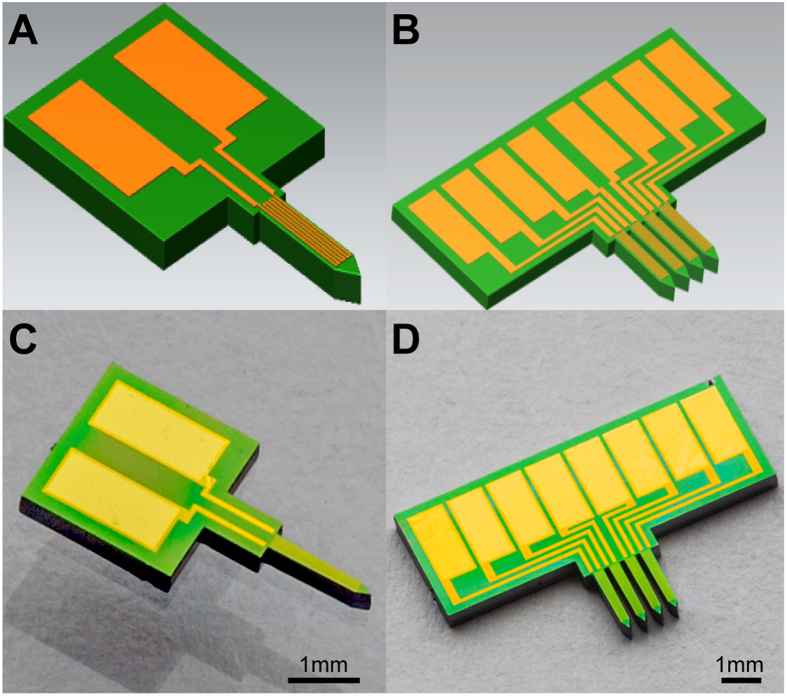
Overview of the presented micro thermal conductivity sensor. **(A)** CAD model of the single-tip micro thermal conductivity sensor. **(B)** CAD model of the multi-tip micro thermal conductivity sensor (four probes). **(C)** Actual single-tip sensor. **(D)** Actual multi-tip sensor (four probes).

The steps involved in fabricating the single probe sensor are shown in Fig. S-1B. Briefly, the fabrication starts with the deposition 500-nm-thick SiO_2_ insulation layer by thermal oxidation on a 400-μm-thick double side polished silicon substrate. It is then followed by the deposition of 15 nm of Cr and 300 nm of Au via a two-step E-Beam evaporation process. The desired serpentine shaped metal layer patterning is afterwards generated with a precision photomask overlay using standard UV photolithography technique (AZ 1512 photo resist) coupled with Au/Cr wet-chemical etch process. To compliment the best resolution range of the digital multi-meter and obtain the most precise resistance readings, the resistance of the developed thermal conductivity sensor is designed to be 500 Ω, which means the total length of the closely packed gold trace (the effective length of the sensing portion) is 22 mm with a 1.5-μm^2^ cross-sectional area; the spacing between traces is 5 μm; the overall geometries of the active sensing region for single tip and multi-tip arrayed sensor are 1.6 mm (depth) by 300 μm (width) and 1.6 mm (depth) by 1.6 mm (width), respectively. The presented multi-tip arrayed sensor has four matching sensing probes with a 150-μm probe-to-probe separation. Subsequently, a very thin insulating dielectric layer of SiO_2_ (100 nm) is deposited on top of the circuits through plasma-enhanced chemical vapor deposition (PECVD) to shield the thin-film metal sensing layer from shortage and corrosive elements in tissue such as saline. Next, another photolithography step (topside alignment, AZ 1512 photo resist) coupled with reactive ion etching process (RIE) are performed to expose the Au soldering pad while keeping the rest of the sensor remain embedded in SiO_2_. Once complete, a third UV exposure step (backside alignment, AZ 1529 photo resist), followed by deep RIE are conducted to etch through the entire substrate, giving the sensors their designated shapes and releasing them from the wafer, as shown in Fig. 5. One should note that the size and the geometry (including the number of tips) of the sensor can easily be reconfigured to accommodate smaller and various shaped samples. Finally, external wiring is achieved using standard cold soldering. Silver conductive epoxy is then applied to welding spots to ensure strong wire linkage.

### Theory, experiment procedure & data analysis protocol

The presented micro thermal conductivity sensor is a miniaturized variation of the previously reported thermal needles with a universal system calibration constant and the fundamental transient hot-wire principle utilized in these thermal probes has been well established for high accuracy simultaneous determination of the thermal conductivity of various liquids, gases and soft materials^6^,^25^,^29^,^30^,^37^. Therefore, the same governing equation for determining thermal conductivity of the measured sample is employed:
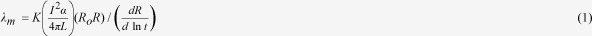
where *λ*_*m*_ is the thermal conductivity of the measuring sample, *K* the system calibration constant, *I* the applied constant current, *α* the temperature coefficient of the sensing material (0.003715 °C^−1^ for pure gold^38^), *L* the effective length of the sensing portion, *R*_*o*_ and *R* the electrical resistance of the metal wire at initial and final temperature *T*_*o*_ and *T*, and *dR*/*d*ln*t* the slope of the linear region in the resistance versus logarithmic time plot, as shown in Fig. S-2.

It is reasonable to believe that material’s thermal properties vary as the temperature changes^1^. This is especially true when investigating thermal conductivity of biological materials as a few degrees of local temperature rise above the physiological norm (37 °C) may result in the enhancement of cell protein expression and abnormal cell behavior, suggesting a state shift from physiological into pathological^4^,^39^. Therefore, keeping the induced local temperature elevation throughout the measurement to a minimum, usually less than 0.5 °C, is vital to ensure unaltered thermal property is measured. In this study, the following measurement procedure and data analysis protocol were adopted^6^ and excised. The testing medium was placed in either a 2.0 mL Eppendorf Safe-Lock microcentrifuge tube (for aqueous samples) or a 60 mm petri dish (for soft biomaterials) inside the temperature regulated thermostatic bath. The samples were left undisturbed and the measurements were commenced only after the sample had reached the targeted temperature and remained steadily. The micro sensor was fully inserted into the sample, and then allowed to equilibrate for 10 min. After the test was initiated, the whole system remained undisturbed for 40 sec. Upon the completion of data acquisition, an automated data analysis and characteristic detection algorithm was performed in MATLAB to identify the most linear region by incrementally screening the entire resistance versus logarithmic time plot (15 ± 5 s time interval with 0.01 s increment), which allows convenient, accurate and objective determination of the slope. The selected linear region should have the highest linear correlation coefficient, *R*^*2*^ (Fig. S-2). Between trials, there was a 10-min cooling off period while the sample was left undisturbed. For experiments when a multi-tip probe was utilized, given that the close proximity between each tip, a prolonged 15-min cooling off time was enforced before the subsequent tip was switched on for measurement.

### Thermal conductivity sensor system calibration & statistical analysis of data

All hot-wire/thermal-needle based thermal conductivity sensors need to obtain system calibration constant in order to correct built-in errors such as approximation or simplified assumptions in the mathematical modeling, imperfections occurred during the device fabrication process, inaccurate measurements from the DMM and small variations in controlling the testing microenvironment. For conventional macroscopic-sized thermal needles, labor-intense system calibration process is performed and repeated for each and every probe. To demonstrate one system calibration fits the entire batch of the presented micro thermal conductivity sensor, distilled water was used as the thermal standard material to obtain the system calibration constant. Furthermore, to examine the generally accepted assumption that the system calibration constant depends solely on the sensor’s geometry and the ambient testing temperature is an insignificant factor, the calibration experiments were conducted from 0 °C to 40 °C.

To ensure data accuracy and reliability in the approach, three randomly selected micro thermal sensors with identical structural and electrical characteristics were utilized and five experiments per condition per probe were performed. Two-way ANOVA test was used to reveal the significant statistical dependence in terms of different sensors and temperature conditions.

### Temperature effect on thermal conductivity of commonly used CPA mixtures & statistical analysis of data

To systematically study the temperature effect on thermal conductivity of common biologically relevant CPA mixtures, 1.5 M and 40% (W/V) G, 1.5 M EG, and 10% (V/V) DMSO were used to conduct an intensive set of experiments at temperatures ranging from −40 °C to 40 °C. Measurement at 4 °C was specifically included in the data set, as it is the temperature that marks the anomalous expansion of water in cryobiology^36^.

Similarly, five experiments per condition per material were performed to ensure good data accuracy and reliability. Each experiment, a randomly selected never-been-used single-tip micro thermal sensor and temperature-specific calibration constants were utilized to determine the thermal conductivity. ANOVA test was used to determine the significant statistical dependence to temperature.

### Soft biological samples with various heterogeneity & statistical analysis of data

To further demonstrate the potential of the developed approach, the arrayed multi-tip sensor was employed to obtain the thermal conductivity distribution of biomaterials with unknown degree of anatomical homogeneity. Experiments were performed on chicken breast, chicken skin, lean porcine limb and bovine liver at 20 °C. For each biological sample, two insertion locations with at least 10 mm of separation were investigated. For each location, one randomly selected freshly prepared multi-tip sensor was utilized in determining the localized thermal conductivity distribution. Similar to previous experimental procedure with the single tip sensor, the multi-tip arrayed micro thermal conductivity sensor was fully inserted into the biological tissues, followed by a brief 5-min hold allowing the undisturbed biomaterials to equilibrate before commencing any actual measurements. A manual switch was used to toggle the DMM testing current between the four tips. Because of the high packing density of the arrayed sensing tips, a prolonged 15-min cooling off time was enforced before the next sequential tip was switched on for measurement.

To maintain high data accuracy and reliability, five repeated trials per four-tip arrayed sensor per location per material were performed. There was also a 15-mm cooling off time between each trial, allowing tissues to be re-conditioned back to its initial state. Results were analyzed by multi-group comparison statistical tests (Two-way ANOVA) to study the statistical significance at both global (between locations) and microscale (between sensing tips).

## Author Contributions

X.M.L., P.K.S., G.Z., Z.Q.S. and D.Y.G. designed the experiments. X.M.L. and P.K.S. fabricated the sensors, conducted the experiments, performed data analysis and interpreted the results. X.M.Z. prepared the CAD drawing of the sensors. X.M.Z., W.P.D., H.Z.P. and Q.C.Z. provided critical inputs to the overall research direction. X.M.L. wrote the manuscript with inputs from all co-authors. All authors reviewed the manuscript. X.M.L. and P.K.S. contributed equally to this work.

## Additional Information

**How to cite this article**: Liang, X. M. *et al.* High accuracy thermal conductivity measurement of aqueous cryoprotective agents and semi-rigid biological tissues using a microfabricated thermal sensor. *Sci. Rep.* **5**, 10377; doi: 10.1038/srep10377 (2015).

## Supplementary Material

Supplementary Information
