# Supplementary material for: High accuracy thermal conductivity measurement of aqueous cryoprotective agents and semi-rigid biological tissues using a microfabricated thermal sensor
Source: Sci Rep. 2015 May 20;5:10377. doi: 10.1038/srep10377 (PMC4438607; doi:10.1038/srep10377)
Supplement: Supplementary Information [file srep10377-s1.pdf]

Title Page

Title: High accuracy thermal conductivity measurement of aqueous cryoprotective agents and semi-rigid biological tissues using a microfabricated thermal sensor

Authors: Xin M. Liang[a,b,c,f,§], Praveen K. Sekar[c,§], Gang Zhao[a], Xiaoming Zhou[d], Zhiquan Shu[c], Zhongping Huang[e], Weiping Ding[a], Qingchuan Zhang[f], Dayong Gao[c]

[a]Centre for Biomedical Engineering, Department of Electronic Science and Technology, University of Science and Technology of China, Hefei, Anhui 230027, China

[b]USTC Center for Micro- and Nanoscale Research and Fabrication, University of Science and Technology of China, Hefei, Anhui 230027, China

[c]Department of Mechanical Engineering, University of Washington, Seattle, WA 98195, USA

[d]School of Mechanical, Electronic, and Industrial Engineering, University of Electronic Science and Technology of China, Chengdu, Sichuan 611731, China

[e]Department of Biomedical Engineering, Widener University, Chester, PA 19013, USA

[f]CAS Key Laboratory of Mechanical Behavior and Design of Material, Department of Modern Mechanics, University of Science and Technology of China, Hefei, Anhui 230027, China

[§]These authors contributed equally to this work.

Correspondence should be addressed to D.Y.G., X.M.L. and G.Z. (dayong@uw.edu; liangxin@ustc.edu.cn; zhaog@ustc.edu.cn)

Supplementary Figures

Supplementary Figure S-1

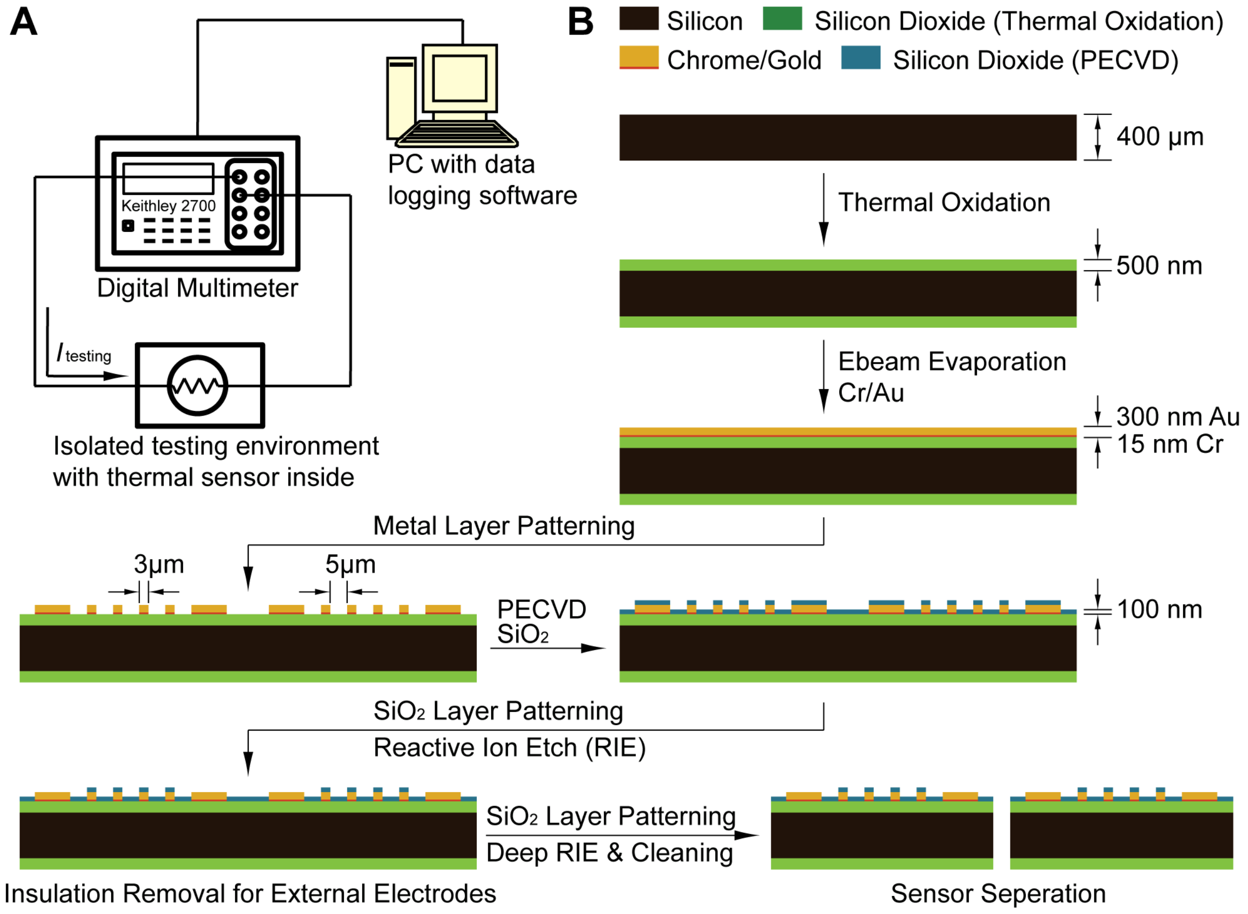

Fig. S-1 Micro thermal conductivity sensor system. **(A)** Schematic of the experimental apparatus for measuring thermal conductivity using the micro thermal sensor. **(B)** Microfabrication flowchart for manufacturing the presented thermal conductivity sensor. The authors are the copyright holder of all drawings presented in this figure. All images are permitted to publish under an Open Access license.

Supplementary Figure S-2

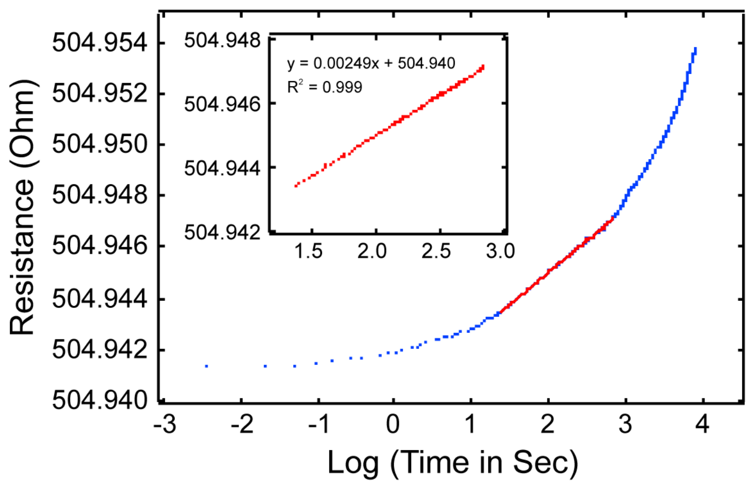

Fig. S-2 Typical electrical resistance response over logarithmic time (overall and close-up view of the most linear region) for glycerol at 20 ºC using the presented micro thermal conductivity sensor.
